# Two-weeks-sustained unresponsiveness by oral immunotherapy using microwave heated cow’s milk for children with cow’s milk allergy

**DOI:** 10.1186/s13223-016-0150-0

**Published:** 2016-08-26

**Authors:** Masaya Takahashi, Shoichiro Taniuchi, Kazuhiko Soejima, Yasuko Hatano, Sohsaku Yamanouchi, Kazuanri Kaneko

**Affiliations:** Department of Pediatrics, Kansai Medical University, 2-5-1 Shin-machi, Hirakata, Osaka Japan

**Keywords:** Food allergy, Microwave heated cow’s milk, Cow’s milk specific IgE, Sustained unresponsiveness, Oral immunotherapy

## Abstract

**Background:**

Several studies have reported that oral immunotherapy (OIT) is effective for children with cow’s milk (CM) allergy. These studies reported the efficacy of OIT in terms of desensitization, but did not describe sustained unresponsiveness to CM. The aim of this study was to evaluate the efficacy of the OIT protocol using microwave heated cow’s milk (MH-CM) in terms of 2-weeks-sustained unresponsiveness (2-weeks-SU) and safety.

**Methods:**

Forty-eight children were enrolled in this study. Thirty-one children agreed to receive rush OIT using MH-CM (the OIT group) and another 17 children who did not want to receive rush OIT formed the untreated group. Rates of desensitization and 2-weeks-sustained unresponsiveness were compared between the two groups at 1 year after the start of OIT. We followed up these rates and safety data for another year and for longer in the OIT group.

**Results:**

No children in the untreated group did not pass an open food challenge to CM. Of the 31 children in the OIT group, 14 (*P* = 0.002) achieved desensitization, and 8 (*P* = 0.036) achieved 2-weeks-SU to CM at 1 year from the start of OIT. Two years after the start of OIT, both the rate of desensitization and the rate of 2-weeks-SU in the OIT group significantly increased compared with the rates at 1 year (*P* = 0.025 and *P* = 0.008 respectively).

**Conclusions:**

The rush OIT protocol using MH-CM was effective at inducing 2-weeks-SU s to CM and had a good safety profile in children with CM allergy.

*Trial registration* Approval number: 324, Registered 3 February 2009

## Background

Cow’s milk (CM) is a common food ingested by children, and is the second most common immediate-type food allergy in Japanese children [1]. Allergen avoidance is the basic approach for the management of food allergy until clinical tolerance is confirmed. Although 50 % of children have tolerance to CM by 5 years of age and increasing to 75 % by their early teenage years [2], some children experience persistent allergic reactions [3, 4]. Oral immunotherapy (OIT) for food allergy has often been used for young children with CM allergy [6–15]. However, efficacy of OIT was only previously described in terms of desensitization and also there are no reports of sustained unresponsiveness and efficacy of following cow’s milk oral immunotherapy (CM-OIT) using microwave heated cow’s milk (MH-CM). Therefore, the true efficacy of OIT for CM allergy is unclear.

Kim et al. previously showed that the addition of baked milk to the diet of children tolerating such foods appears to accelerate the development of unheated-milk tolerance compared to strict avoidance [16]. An explanation of this observation might be that the high temperature reduces allergenicity by destroying conformational epitopes of milk proteins that subsequently it promotes unheated CM tolerance. Another recent study reported that MH-CM reduced allergic responses in a mouse allergic model [17].

No study has been performed to investigate OIT for children with CM allergy using MH-CM. The aim of the present study was to evaluate the sustained unresponsiveness and safety of an OIT protocol using MH-CM in children with CM allergy compared with an untreated group.

## Patients and methods

### Study design

A prospective, longitudinal, intervention study was performed from June 2010 to March 2015 in the Department of pediatrics of Kansai Medical University Hospital, Osaka, Japan. The study protocol was not registered in the official internet registration system in Japan, but was approved by the Institutional Ethics Committee of Kansai Medical University, which included defining the outcomes prospectively.

### Patients

Inclusion criteria were as follows: aged 5–18 years and persistent CM allergy within the 2 weeks before the start of CM-OIT that was confirmed by (1) positive clinical history, (2) positive food challenge test to CM and (3) level of CM specific immunoglobulin E (sIgE, CAP-Phadia, Uppsala, Sweden) to CM above 0.35 kUA/L. We ensured that families had adequate information regarding the study and understood the implications of participation. Signatures designating informed consent were obtained. Exclusion criteria were as follows: severe atopic dermatitis and uncontrolled asthma (baseline FEV_1_ < 80 % of predictive value) according to Japanese pediatric guideline for the treatment and management of bronchial asthma [18].

We enrolled 48 children who met these criteria. Those who agreed to treatment were assigned to the treatment group (OIT group), and those who did not want to receive treatment were assigned to the control group (the untreated group). This allocation was not done in a random manner and was based on the decision of the children and parents. The patients in the untreated group were followed up at 10–14 months, and oral food challenge (OFC) and blood examinations were repeated.

### Oral food challenge

All CM challenges were open challenges, and were performed at hospital settings and supervised by physicians. Clinical features of a reaction to CM were investigated for clinical purposes via an OFC as described previously [19]. A double-blind placebo-control food challenge is the gold standard for clinical studies, but is time-consuming in general practice. We could not assess subjective symptoms by OFC. Therefore, if patients had subjective allergic symptoms such as nausea, abdominal pain, sore throat, or itching, we increased the loading dose until the objective symptoms appeared. During the challenge, full emergency equipment was at hand. Prior to enrollment in the study, the children’s parents provided informed consent. Patients were asked to avoid anti-histamine use for 72 h before the OFC, but topical steroids were allowed. Patients were admitted to our day clinic in the morning. The challenge material for the OFC was fresh CM. The procedure is described in detail in Table 1. The challenge was interrupted if children demonstrated unambiguous clinical reactivity or after the administration of 40 mL of CM. Following the cessation of CM feeding, all children were observed for at least 3 h. In cases of obvious allergic symptoms (such as rash, coughing, vomiting, or diarrhea) to loading doses of less than 40 mL CM positivity was indicated; otherwise, CM negativity was indicated. The threshold dose was defined as the last dose in OFC at which only objective symptoms occurred and the levels of severity of allergy to CM were assessed by Sampson’s grading score [20].

**Table 1:** Oral food challenge and Rush OIT protocol

	*Oral food challenge*
Procedure	Open food challenge
Materials	Fresh CM or MH-CM (heated for 100 s in 550 W microwave oven) or
Initial dose	0.1 mL 1 mL (if no anaphylaxis is suspected)
Subsequent dose	0.2, 0.5, 1,2, 5. 10, 20 and 40 mL 2, 5, 10, 20 and 40 mL (if no anaphylaxis is suspected)
Interval	Every 30 min
Threshold dose	Defined as the last dose in OFC at which only objective symptoms occurred and the levels of severity of allergy to CM [20]
*Rush OIT*	
Initial dose	Starting approximately at a tenth of the threshold dose determined at OFC using MH-CM 1.2-fold for each patient
Increase in dose	Starting with MH-CM, the dose was increased approximately 1.2-fold every time Ingest MH-CM 2–4 times a day at 2-h intervals If grade^a^ 2 or 3 develops, the same dose or the previous tolerated dose was repeated If grade^a^ 4 develops, OIT was stopped and the previous tolerated dose was repeated on the next day When reaching 200 mL of MH-CH, the ingestion was once a day No further increases in dosage because of repeated adverse events, after the highest tolerated dose was continued for 3 consecutive days without any allergic reaction, the rush OIT was terminated
Maintenance	Ingest the maintenance dose of 200 mL of MH-CM everyday for maintenance at home If the subject did not reach the target dose of 200 mL of MH-CM, the loading dose was gradually increased by 1 mL per day until the target dose of 200 mL was reached and the dose was continued
MH-CM to fresh CM	In cases where no adverse reactions were observed for 2 months during a daily intake of 200 mL of MH-CM, the time spent heating the CM in the microwave oven was gradually shortened by 10 s every week

*OIT* oral immunotherapy, *CM* cow’s milk, *OFC* open food challenge, *MH-CM* microwave heated cow’s milk

^a^Grade of anaphylaxis according to Sampson’s score [20]

We also used MH-CM for OFC before OIT to define the initial dose of MH cow’s milk oral immunotherapy. Fresh CM was warmed in a microwave oven at 550 W for 100 s and then cooled to room temperature before the OFC. The MH-CM food challenge was only performed with the OIT group immediately before the start of OIT.

### Oral immunotherapy

OIT was performed within 2 weeks of the OFC using fresh CM. All the patients were admitted to our hospital. The OIT was performed following a previously described method [19]. The OIT protocol is described in detail in Table 1. The OIT consisted of two phases: (1) a rush phase, and (2) a maintenance phase.

After patients had undergone the maintenance phase followed by the rush phase, we evaluated whether patients in the OIT group were desensitized or had sustained unresponsiveness to CM at 1-, 2-, 3- and 4-year follow-up. When patients ingested 200 mL of fresh CM daily for 2 months without adverse events, they were considered desensitized to CM. If there were no adverse events for 2 months with a daily intake of 200 mL of fresh CM, a CM-OFC was performed at 2 weeks after discontinuation of the daily administration of 200 mL of fresh CM. During the 2 weeks the patient continues strictly elimination of CM products. Briefly, the CM-OFC was undertaken using the total 200 mL of fresh CM. The initial dose was set at 15 mL, and the increased at 30, 60 mL, and finally 95 mL (total 200 mL) every 30 min. If the result of the CM-OFC was negative, the patient was defined as having 2-weeks-sustained unresponsiveness (2-weeks -SU) for CM. Additionally, after the positivity of CM-OFC, the patient continued a daily intake of 200 mL of fresh-CM. After patients showed, 2-weeks-SU, the patients were free to ingest CM or CM-containing products.

Twenty of the OIT patients were followed for 4 years after the initiation of CM-OIT and 30 were followed for 3 years. In comparison, in the untreated group, patients continued the elimination diet of CM and CM products after their OFCs. One year after the initial OFC, OFC with fresh CM was performed for the untreated group, and the results were compared with their previous OFC results.

### Laboratory tests

Blood samples were collected before starting the rush OIT and at 1 and 2 years after the start of OIT.

### Analyses

Two statistical analyses were performed: (1) comparison of the rates of desensitization and 2-weeks-SU at 1 year in the OIT and the untreated groups at 1 year, and (2) comparison of the rates of desensitization and 2-weeks-SU in the OIT group at 1- and 2-year follow-ups. Furthermore, we statistically analyzed the same factors indicated in Table 2 among children in the OIT group, to determine whether the baseline characteristics differed between the patients who achieved 2-weeks-SU to 200 mL of fresh CM at 2 years after the start of OIT and those who failed to achieve 2-weeks-SU.

**Table 2:** Baseline characteristics of patients in the study group

Characteristic	Group		*P* value
	OIT (N = 31)	Untreated (N = 17)	
Gender (male)	23	14	0.396
Age at the challenge test			
Median	9	7	0.179
Range	(5–17)	(5–16)	
Initial total IgE level (IU/mL)			
Median	871	935	0.940
Range	(90–21966)	(122.7–30725)	
Initial CM-specific IgE level (kUA/L)			
Median	25	29	0.407
Range	(0.9–5730)	(0.56–6290)	
Initial casein-IgE level (kUA/L)			
Median	21.5	28.4	0.693
Range	(1.1–7210)	(1.12–8350)	
Initial β-lactoglobulin-IgE level (kUA/L)			
Median	2	1.05	0.425
Range	(0–61.8)	(0.05–75.3)	
Presence of other food allergies^a^			
No	10 (32 %)	3 (18 %)	0.229
Yes	21 (68 %)	14 (82 %)	
Frequency of anaphylaxis at accidental ingestion			
Never	9 (29 %)	2 (12 %)	0.109
Once	12 (39 %)	6 (35 %)	
2–10 times	10 (32 %)	9 (53 %)	
>10 times	0	0	
Atopic dermatitis	13	18	0.48
Asthma			
Severity of asthma^a^	14 (80 %)	10 (59 %)	0.376
Intermittent	3 (45 %)	2 (12 %)	
Moderate	11 (35 %)	8 (47 %)	
Persistent	0	0	
Threshold dose of CM at OFC (mL)			
Median	2	2	0.337
Range	(0.1–40)	(0.5–10)	
Grade of allergic reaction at OFC^b^			
1	8 (26 %)	2 (12 %)	0.823
2	13 (42 %)	12 (71 %)	
3	6 (19 %)	3 (18 %)	
4	4 (13 %)	0 (0 %)	

*CM* cow’s milk, *OIT* oral immunotherapy, *OFC* open food challenge

^a^ Severity of asthma according to Japanese Pediatric Guideline for the treatment and management of bronchial asthma [21]

^b^ Grade of anaphylaxis according to Sampson’s score [20]

### Statistics

We statistically evaluated the clinical outcome of the OIT group (31 children) and the untreated group (17 children) at 1 year, using a 2-sided alpha level of 0.05, to detect a significant difference between the 2 groups. Using Fisher’s exact test and the Mann–Whitney U-test tested baseline characteristics of the patients. Fisher’ exact test was used to evaluate between-group differences with regard to achieving desensitization and 2-weeks-SU to 200 mL of CM at 1-year follow-up. We also evaluated the differences in achieving a remission stage at the 1- and 2-year follow-ups within the OIT group using the Wilcoxon sign test. All analyses were performed with Excel Statcel 3 (Microsoft Inc. USA).

## Results

### Baselines characteristics

The baseline characteristics of these subjects are shown in Table 2; no significant differences were noted between the 2 groups.

### Clinical outcomes

**Fig. 1:**
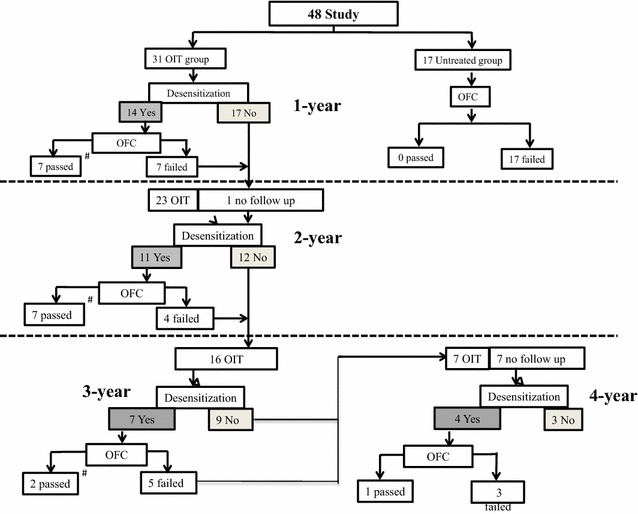
Study enrollment and outcomes of *OIT* oral immunotherapy with *CM* cow’s milk. *(Hash)* After the achievement of 2-weeks-sustained unresponsiveness, the patients are followed for 2–3 year and they are able to ingest CM and CM products freely without any adverse events. *OFC* open food challenge

Study enrollment and outcomes are shown in Fig. 1 and Table 3. Fourteen of 31 children (42 %) in the OIT group were categorized as desensitized to fresh CM at the 1-year follow-up, whereas none in the untreated group passed an oral food challenge. In addition, there was a statistically significant difference between the 2 groups (*P* = 0.002). In the untreated group, the threshold doses (median 1 mL and range 0. 5–10 mL) and severity levels of allergy (median 2 and range 1–3) to CM in the initial OFC was not significantly different to the threshold doses (median 1.75 mL and range 0.1–40 mL) and severity levels of allergy (median 2 and range 1–4) in the second OFC (*P* = 0.913, *P* = 0.688), respectively. The repeated OFC in the untreated group was performed at the 1-year interval (median: 12 months and range: 10–14 months). In addition, 7 of 31 children (21 %) in the OIT group showed a significant induced 2-weeks-SU to CM in contrast to 0 of 17 children in the untreated group at 1-year follow-up (*P* = 0.036) (Table 3). By the 2-year follow-up, both the rates of desensitization and 2-weeks-SU sustained in the OIT group were significantly increased compared with the rates at the 1-year follow-up (*P* = 0.025 and *P* = 0.008, respectively, by Wilcoxon rank test). Both the rates of desensitization and 2-weeks-SU gradually increased every year and reached 85 and 70 %, respectively, by 4 years (Table 3). Two to 3 years after achieving, 2-weeks-SU, we followed the patients and confirmed that the patients did not experience any adverse events including oral itch after the ingestion of CM or CM products, including CM dependent exercise-induced anaphylaxis. Patients did not experience any adverse events in the follow-up period.

**Table 3:** The rate of desensitization and 2-weeks-sustained unresponsiveness for cow’s milk allergy in the oral immunotherapy group and the untreated group

	OIT (N = 31)		Untreated group (N = 17)
	Desensitization	Two-weeks-sustained unresponsiveness	Pass OFC
At 1-year follow-up	14/31 (45 %)*^, #^	7/31 (21 %)**^, ##^	0/17 (0 %)^a^
At 2-year follow-up	18/30 (60 %)^#^	14/30 (47 %)^##^	
At 3-year follow-up	21/30 (70 %)	16/30 (53 %)	
At 4-year follow-up	17/20 (85 %)	14/20 (70 %)	

*OIT* oral immunotherapy, *OFC* open food challenge

* *P* = 0.002 by Fisher’s exact test

** *P* = 0.036 by Fisher’s exact test

^#^ *P* = 0.025 by Wilcoxon signed rank test

^##^ *P* = 0.008 by Wilcoxon signed rank test

^a^Patients in untreated groups continued complete elimination of cow’s milk and a year later were performed open food challenge using fresh cow’s milk was performed

The levels of CM-sIgE decreased significantly in the OIT group (median CM-sIgE level, 25.0–12.2 kUA/L) during 1-year follow-up. The levels of CM-sIgE also decreased in the untreated group (median CM-sIgE level, 29.2–27 kUA/L) between the initial OFC and the OFC at 1 year-interval.

### Threshold dose and grade of anaphylaxis using MH-CM in OFC

**Fig. 2:**
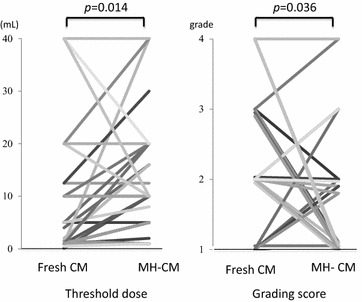
Changes of the threshold dose and the severity of the grade of anaphylaxis by Samson’s grading score [20] at *OFC* open food challenge using fresh *CM* cow’s milk and microwave heated cow’s milk (MH-CM). The MH-CM food challenge was only performed with the OIT group at the start of OIT. Fresh CM was heated in a microwave oven at 550 W for 100 s and the cooled to room temperture before the OFC

Before the rush OIT using MH-CM, OFC using MH-CM was performed. The threshold dose (median 10 mL and range 1–40 mL) was significantly higher for patients using MH-CM than for the patients using fresh CM (median 2 mL and range 0.1–40 mL) (*P* = 0.014) (Fig. 2). In addition, the severity of the grade of allergic reaction was milder for patients who received MH-CM compared with that for patients receiving fresh CM (*P* = 0.036) (Fig. 2).

### Skin prick test

Before the rush OIT, a skin prick test (SPT) was performed in 10 of 21 patients in the OIT group using MH-CM and fresh CM. The median wheal size of SPT (median 10.5 mm and range 7–16 mm) using MH-CM was not statistically different compared with that of SPT using fresh CM (median 11.5 mm and range 7–16 mm) (*P* = 0.878 by Wilcoxon rank test).

### Baseline characteristics in the OIT group according remission and continued intolerance

There were no significant differences of threshold dose and severity of the grade of allergic reaction to CM at OFC between the patients who achieved 2-weeks-SU and those who failed to achieve 2-weeks-SU by the 1-year follow up. However, the following factors were significantly different between the patients who achieve 2-weeks-SU and those who did not (Table 4): initial serum CM-sIgE level (*P* = 0.0006), casein-sIgE level (*P* = 0.0005), β-lactoglobulin-sIgE level (*P* = 0.017), and severity of asthma *(P* = 0.03).

**Table 4:** Baseline characteristics of patients in the intolerance group and the 2-weeks -sustained unresponsiveness group

Characteristic	Group		*P* value
	Intolerance (N = 17)	Two-weeks-sustained unresponsiveness (N = 14)	
Gender (male)	12	10	0.637
Age at the challenge test			
Median	7	10	0.469
Range	(5–17)	(6–15)	
Initial total IgE level (UA/mL)			
Median	1016	497	0.165
Range	(202–21,966)	(90–6337)	
Initial CM-specific IgE level (kUA/L)			
Median Range	87.4 (0.9–5730)	5.88 (1.78–55)	<0.001
Initial casein-IgE level (kUA/L)			
Median	83.9	7.445	<0.001
Range	(1.1–7210)	(1.12–86.9)	
Initial β-lactoglobulin-IgE level (kUA/L)			
Median	8.6	1.66	0.017
Range	(0.2–23.8)	(0.0–17.4)	
Presence of other food allergies^a^			
No	5 (29 %)	5 (36 %)	0.503
Yes	12 (71 %)	9 (64 %)	
Frequency of adverse events/child/dose (%)			
Total events	17	7.3	0.503
Grade 1	8	3.6	
Grade 2	3.6	0	
Grade 3	2.4	0	
Grade 4	0	0	
Atopic dermatitis	3 (18 %)	1 (7 %)	0.378
Asthma Severity of asthma^a^			
Intermittent	1 (6 %)	3 (21 %)	0.030
Moderate	9 (53 %)	7 (7 %)	
Persistent	0	0	
Threshold dose of fresh CM at OFC (mL)			
Median	1.5	5	0.790
Range	(0.1–40)	(1–20)	
Threshold dose of heated CM at OFC (mL)			
Median	10	15	0.091
Range	(1–40)	(5–40)	
Grade of allergic reaction at OFC (fresh CM)^b^			
1	3 (18 %)	5 (36 %)	0.415
2	7 (41 %)	6 (43 %)	
3	3 (18 %)	3 (21 %)	
4	4 (23 %)	0 (0 %)	
Grade of allergic reaction at OFC (heated CM)^b^	8 (41 %)	9 (64 %)	0.415
1	6 (35 %)	3 (21 %)	
2	2 (12 %)	1 (7 %)	
3	1 (6 %)	1 (7 %)	

*CM* cow’s milk, *OFC* open food challenge

^a^Severity of asthma according to Japanese Pediatric Guideline for the treatment and management of bronchial asthma [21]

^b^Grade of anaphylaxis according to Sampson’s score [20]

### Safety data during the OIT period

Table 5 shows the average frequencies of adverse events that occurred per dose in each subject either at hospital or at home. In the rush phase of OIT (in hospital), the average of adverse events per subject per dose was 0.167. According to Sampson’s classification [20], 0.113 of subjects had grade 1 adverse events, and 0.035 was grade 2, 0.017 were grade 3 and 0.002 was 4. At home during the following year, the average of adverse events per subject per dose was 0.085 and 0.041 of subjects had grade 1 adverse events, 0.035 were grade 2, 0.01 were grade 3 or 0.0004 were 4. At home between years 1 and 2, the average of adverse events per subject per dose was 0.044 and 0.024 of subjects had grade 1 adverse events, 0.013 were grade 2, 0.0005 were grade 3 or 0.0002 were 4.

**Table 5:** Averages of reactions and treatments per dose per child in the rush phase of oral immunotherapy (in hospital), at 1 year (at home) and at 1–2 years (at home)

Reactions per dose per child					
	Total	Grade 1^a^	Grade 2^a^	Grade 3^a^	Grade 4^a^
Rush phase	0.167	0.113	0.035	0.017	0.002
1 year	0.085	0.041	0.035	0.01	0.0004
1–2 years	0.044	0.024	0.013	0.0005	0.0002

Treatments per dose per child						
	Anti-histamines	Nebulized epinephrine	Nebulized β_2_ agonists	Oral steroids	Intravenous steroids	Epinephrine injection
Rush phase	0.039	0.02	0.02	0.04	0.003	0.003(6)^b^
1 year	0.02	0	0.017	0.027	0.0009	0.0006(9)^b^
1–2 years	0.01	0	0.005	0.02	0	0.0001(1)^b^

Each data express averages of reactions and treatments per dose per child

^a^Grade of anaphylaxis according to Sampson’s score [20]

^b^Total numbers of injection

For the treatment of these symptoms, 21 of 31 (64 %) children received medication at hospital. The averages of those receiving treatments per subject per dose are shown in Table 2. The number of patients receiving oral anti-histamines nebulized β-2 agonists, nebulized epinephrine agonists, oral dexamethasone, and intravenous steroids gradually decreased over the 2 study years. However, in the intolerant group, mild and moderate adverse events occurred and were treated by anti-histamines or nebulized β-2 agonists. Three patients each received a single epinephrine injection and one received three epinephrine injections at hospital. Four patients each received a single epinephrine injection, one received two epinephrine doses and one received three doses of epinephrine at the 1-year follow-up. In addition, these children did not achieve desensitization by 2 years after the start of OIT.

## Discussion

The present study described the efficacy and safety of OIT using MH-CM for children with severe CM allergy compared with an untreated group. Three randomized control trials of CM-OIT previously reported significant differences of the threshold dosage and desensitization rates between patients who underwent OIT and those who maintained an elimination diet [5, 8, 9]. The success rate for desensitization ranged from 36 to 67 %. In our study, the desensitization rates were 45, 60, 70 and 85 % at 1, 2, 3 and 4 years after starting OIT, respectively. The three previous studies did not describe the results of CM sustained unresponsiveness [5, 8, 9], although one study described the rate of sustained unresponsiveness CM-OIT [21] in which 75 % of patients were successfully desensitized; however, patients who passed the OFC 2 weeks after ceasing the OIT included 27.1 % of patients on CM and those patients might have achieved sustained unresponsiveness. The published rates for sustained unresponsiveness are similar to those obtained in our study, but the rates of achieving desensitization were higher than in our study. This difference might be explained by the different protocols used or by the different study populations.

The period following the discontinuation of OIT was relatively short (2 weeks) to assess sustained unresponsiveness. Although this approach was selected to be in line with NIAID–FDA recommendations food allergy clinical trial design at the time the study was designed and registered [22], it is acknowledged that a longer period of at least 4 weeks after discontinuation of treatment would now be advised. We plan to perform a longer period follow-up study (5 or more years after OIT) in which subjects will undertake an OFC after 4–8 weeks of secondary CM elimination to assess prolonged sustained unresponsiveness.

MH-CM is prepared by heating in a microwave oven for 100 s. The temperature of MH-CM is almost 60 °C and is not aggregated. Before the intake of CM, MH-CM was cooled to room temperature. The threshold for MH-CM-OFC significantly increased compared with that of fresh CM-OFC. In addition, the severity of allergic symptoms in the MH-CM-OFC was significantly less than that for the fresh CM-OFC. Three children had a decreased threshold to MH-CM, two of which showed decrease in their grading score (4-1 and 2-1). The third child showed no change in grading score. The grading score of the child receiving fresh CM and MH-CM had scores of 2 and 2, respectively, and showed sporadic cough. A reason why there was no difference in grading score and decreased threshold to MH-CM between the two OFCs might be unclear.

In a study by Mecherfli et al. to examine whether the observed microwave effects were dependent upon temperature, peptic hydrolysis was studied with conventional heating using the same temperatures as those attained with different microwave wattages: 43.2, 51.7 and 63.5 °C for 50 W (5 min), 100 W (3 min) and 200 W (3 min) irradiation, respectively [17]. They found a significant effect on peptic hydrolysis of purified β-lactoglobulin at 200 W (3 min) with 52 % protein degradation as estimated by electrophoretic densitometry relative to conventional heating at an identical temperature. Thus, microwave effects on peptic hydrolysis were significant compared with conventional heating to the same temperature. We measured the temperature of MH-CM at 550 W for 100 s, and found the value to be 60 °C. The effect on our microwave-heated method at 550 w (100 s) was supposed to be equal to that of the method at 200 w (3 min). Mecherfli et al. also confirmed this hydrolysis effect by pepsin using the sera of young patients allergic to bovine whey proteins using an anti-IgE immunoblotting assay [23]. These results suggest that microwave-heated treatment reduces the allergenicity of cow milk proteins containing β-lactoglobulin by enzymatic digestion in the intestine. Therefore, in the MH-CM-OFC, patients had higher threshold doses and fewer allergic symptoms than those in the fresh CM-OFC, though there were no significant differences in the wheal diameter by SPT between heated CM and fresh CM groups. A significant difference between the two OFCs was observed, but this was small. Therefore, future investigations should use a larger number of the two OFCs or double blind placebo control study of OIT using both MH-CM and fresh CM to elucidate whether MH-CM is a better intervention than fresh CM.

Adverse events in the MH-CM-OIT group were also mild. It is difficult to compare the frequencies of adverse events during OIT with other studies because the methods of calculating adverse reactions and the classification of adverse reactions are different among studies. The only way to assess the adverse events is by using the percentage of patients requiring epinephrine. The percentage of patients requiring epinephrine in previous studies was 6.7 [6], 13.3 [8] 16.7 [5], and 30.8 % [9], whereas in our study it was 12.9 %. The two studies that had lower rates of epinephrine usage [6, 8] enrolled different age populations and use a different dosing method compared with our study. The study design and patient populations of the two studies with higher rates of epinephrine usage [5, 9] were similar to those of our study. The percentages of epinephrine treatment in these two studies were higher than that in our study. Based on these findings, MH-CM-OIT may be relatively safe compared with fresh CM-OIT.

We statistically analyzed the factors in Table 4 for children in the OIT group to determine whether there was a difference in baseline characteristics between the patients who achieved 2-weeks-SU to 200 mL of fresh CM and those who failed to do so. There were several significant differences between the patients who achieved 2-weeks-SU and those who failed to achieve 2-weeks-SU including initial serum CM-sIgE level (*P* = 0.0006), casein-sIgE level (*P* = 0.0005), β-lactoglobulin-sIgE level (*P* = 0.017), and severity of asthma (*P* = 0.03) (Table 2). Similar results using fresh CM-OIT protocol were reported by Vázquez-Ortiz et al. [24]. Although they analyzed the baseline characteristics of the two groups by safety data. Cox proportional hazard multivariate regression model identified three variables (CM-sIgE, CM-skin prick test and Sampson’s severity grades at baseline food challenge) as independent risk factors for the persistence of allergic reactions. It is very interesting to note that the factors predicting prognosis are serum CM-sIgE level than the threshold dose by OFC.

There were several limitations in our study. First, the study was not randomized. The observational period of the untreated group was limited to 1 year and we could not extend it because all the patients wanted to start either slow-type home-based OIT or rush type OIT after 1-year of observation. Therefore, we could not set a control group for the whole study period. Second, the immunological markers assayed were limited to IgE levels, and we were not able to include serum IgG4 levels or the SPT. Third, multiple regression analysis was used to identify independent factors to predict tolerance and reactivity. However the sample size was too small to determine whether the factors were significant. Fourth, the OIT and untreated groups are not equivalent in terms of their demographics, because there was a trend in the control group toward increased atopy (slightly higher total and CM-sIgE levels, increased percentage with other food allergies, increased percentage with moderate asthma), an increased percentage who had multiple prior episodes of anaphylaxis after accidental ingestion, and an increased percentage with grade 2 or higher anaphylaxis during initial OFC. These factors may be considered to cause a self-selection bias in the untreated group. Finally, we already had performed fresh CM-OIT in ten children before the start of the present study. We could not compare the study group using fresh CM-OIT, because the study was different from the present protocol. However, our study is the first study to assess 2-weeks-SU in CM-OIT compared with a control group using a MH-CM material.

## Conclusions

In this study, we found that a rush OIT protocol using MH-CM was effective in a significant percentage of children with CM allergy and showed a good safety profile. Furthermore, serum CM-sIgE levels might be useful to predict 2-weeks-SU and safety of CM-OIT.

## Abbreviations

CM: Cow’s milk
OIT: oral immunotherapy
MH-CM: microwave heated cow’s milk
2-weeks-SU: 2-weeks-sustained unresponsiveness
sIgE: specific immunoglobulin E
OFC: oral food challenge
SPT: skin prick test
