# Erratum to: Two-weeks-sustained unresponsiveness by oral immunotherapy using microwave heated cow’s milk for children with cow’s milk allergy

**DOI:** 10.1186/s13223-016-0160-y

**Published:** 2016-10-31

**Authors:** Masaya Takahashi, Shoichiro Taniuchi, Kazuhiko Soejima, Yasuko Hatano, Sohsaku Yamanouchi, Kazuanri Kaneko

**Affiliations:** Department of Pediatrics, Kansai Medical University, 2‑5‑1 Shin-machi, Hirakata, Osaka Japan

## Erratum to: Allergy Asthma Clin Immunol (2016) 12:44 DOI 10.1186/s13223-016-0150-0

After publication of the original article [1], the author noticed that there are some error with Table 3 and Fig. 1. The corrected Table 3 and Fig. 1 is given in this erratum. We apologize for any inconvenience this may cause.

**Table 3:** The rate of desensitization and 2-weeks-sustained unresponsiveness for cow’s milk allergy in the oral immunotherapy group and the untreated group

	OIT (N = 31)		Untreated group (N = 17)
	Desensitization	Two-weeks-sustained unresponsiveness	Pass OFC
At 1-year follow-up	14/31 (45 %)*^, #^	7/31 (21 %)**^, ##^	0/17 (0 %)*^,^**^, a^
At 2-year follow-up	18/30 (60 %)^#^	14/30 (47 %)^##^	
At 3-year follow-up	21/30 (70 %)	16/30 (53 %)	
At 4-year follow-up	17/20 (85 %)	14/20 (70 %)	

*OIT* oral immunotherapy, *OFC* open food challenge

* *P* = 0.002 by Fisher’s exact test

** *P* = 0.036 by Fisher’s exact test

^#^ *P* = 0.025 by Wilcoxon signed rank test

^##^ *P* = 0.008 by Wilcoxon signed rank test

^a^Patients in untreated groups continued complete elimination of cow’s milk and a year later were performed open food challenge using fresh cow’s milk was performed

**Fig. 1:**
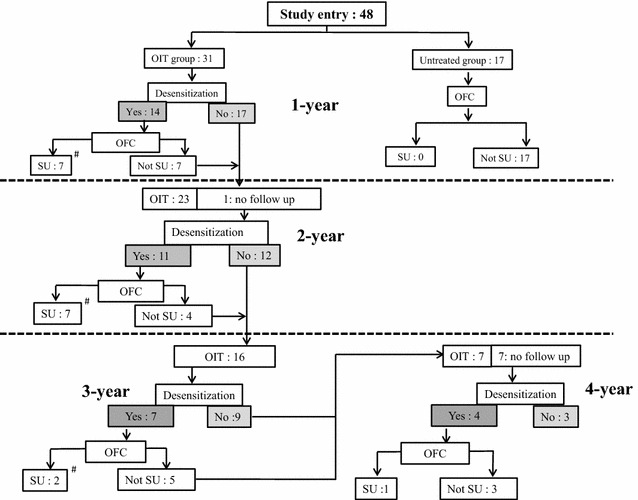
Study enrollment and outcomes of *OIT* oral immunotherapy with *CM* cow’s milk. *(Hash)* After the achievement of 2-weeks-sustained unresponsiveness, the patients are followed for 2–3 year and they are able to ingest CM and CM products freely without any adverse events. *OFC* open food challenge
